# Valuing Place in Rural Health Education and Research: The Contribution of University Departments of Rural Health

**DOI:** 10.1111/ajr.70209

**Published:** 2026-05-13

**Authors:** Pam Harvey, James Debenham, Joanne Hutchinson, Leanne Brown

**Affiliations:** ^1^ Monash Rural Health Monash University Bendigo Victoria Australia; ^2^ Majarlin Kimberley Centre for Remote Health The University of Notre Dame Broome Western Australia Australia; ^3^ Australian Rural Health Education Network Canberra Australian Capital Territory Australia; ^4^ Department of Rural Health University of Newcastle Tamworth New South Wales Australia

## Abstract

**Aims:**

This commentary argues that University Departments of Rural Health (UDRHs) are integral to shaping distinct, contextually grounded value for rural communities and healthcare systems through their programs of place‐based education and research.

**Context:**

For over two decades, the Rural Health Multidisciplinary Training (RHMT) Program has addressed rural healthcare inequities through a place‐based approach to health education and research. UDRHs, funded under the RHMT Program, deliver academic activities for nursing, midwifery and allied health students and clinicians, including placements, promoting rural health careers and conducting research relevant to their regions.

**Approach:**

This commentary draws on recent evidence of the value of place‐based training and research for rural Australia. Place‐based education is where the integration of concepts such as connection to and engagement with place supports the preparation of health professions students for the complexity of rural healthcare. Place‐based research contributes contextually situated evidence that informs the ongoing development of healthcare education and service delivery. Together, these concepts can be understood as shaping the conditions for high quality, rurally relevant program delivery.

**Conclusion:**

The RHMT Program remains a cornerstone of rural health workforce policy. Its renewal is likely to reinforce the value of contemporary educational, clinical, and research practices for the benefit of people living in rural Australia. Extending the role of UDRHs to target retention strategies has the potential to increase connection and responsiveness to the healthcare needs of rural communities.

## Key points

1. UDRHs create value through place‐based rural health education, not only through placement activity.
2. Place‐based research is a core mechanism for generating rurally relevant knowledge.
3. UDRHs are rural academic centres that contribute to community capital and are well positioned to support early career transition to rural practice.

## 1 Introduction

For over two decades, the Australian Government has supported health professions training to grow and sustain the rural health workforce through the Rural Health Multidisciplinary Training (RHMT) Program. This national network of rurally based academic centres provides opportunities for health students and professionals to study, practice, and build their careers in rural Australia, where 30% of the population resides [1]. The most recent evaluation of the RHMT program describes it as a mature and effective initiative that has “established a university presence in rural communities across Australia, built new capital infrastructure, developed local academic and professional networks, enabled teaching and supervision of students beyond the confines of the city, developed rural research capacity and expertise and strengthened clinical service delivery across Australia*”* [2].

Within the RHMT program, University Departments of Rural Health (UDRHs) deliver a suite of place‐based enablers for nursing, midwifery and allied health students and existing clinicians. Activities include facilitating clinical placements, promoting rural health careers, providing professional development for rural practitioners and conducting research into training, workforce, and health issues relevant to their regions. A priority for UDRHs is working with First Nations communities to ensure health education and research are culturally appropriate, aligned with local priorities and supportive of the First Nations health workforce.

This commentary argues UDRHs are integral to shaping distinct, contextually grounded value for rural communities and health systems through integrated programs of place‐based education and research within the RHMT framework with locally responsive, experience‐based learning and knowledge translation through community and health partnerships. In this context, ‘value’ is understood as multi‐dimensional, encompassing workforce development, research relevance, system improvement and benefits to diverse stakeholders, including government, health services, aged care and disability sectors, universities, students and rural communities. ‘Place‐based education’ is a concept that integrates connection to and engagement with rural locations to create locally responsive learnings [3], valuing experiential learning processes, contextual relevance and practice‐readiness as key outcomes in preparing students for the complexity of rurally based practice. ‘Place‐based research’ is an approach to inquiry that contributes contextually situated knowledge through engagement with local communities, health services and social practices, and is inclusive of geographical location and the everyday practices, perceptions and expectations of the population. Together, these practices position UDRHs as generative sites for the development and application of rural health capabilities, reinforcing their distinct value within the RHMT framework.

## 2 Place‐Based Training for Rural Workforce Generation

The need for a dedicated training system that inspires health careers and equips rurally based professionals to thrive in rural practice remains critically important and is central to the value generated by UDRHs through place‐based training. Evidence indicates that educational strategies such as preferential selection of rural‐origin students, distributed training in rural areas, and opportunities to develop a connection to place are associated with higher rates of rural workforce recruitment [4]. Rural‐origin students are reported to be more likely to undertake multiple rural placements and accumulate greater exposure, factors that have been linked to subsequent rural recruitment and retention [5, 6] There is also evidence to suggest high quality, immersive rural training experiences can influence the professional identity of non‐rural origin students and challenge urban‐normative assumptions, which may contribute to an increased likelihood of rural practice [7, 8].

The concept of place‐based training aligns closely with the Australian Government's *Universities Accord*, which emphasises equity, diversity, and accessibility in higher education [9]. In particular, rurally situated, place‐based health education supports the participation of students living in rural areas [10] and those with First Nations backgrounds [11], groups that have historically been underrepresented in both higher education and the health workforce. This is assisted by providing locally accessible training pathways, culturally responsive learning environments, and supervision models that are grounded in community and cultural context. By being embedded in rural communities, the UDRHs collaborate with rural communities, local schools and vocational education providers to assist students to experience rural life and practice, nurture interest in health careers, establish study pathways and tailor academic support for rural and First Nations students, reducing geographic, cultural and structural barriers to engagement in health education.

Given the complexities of delivering rural healthcare, health training must remain dynamic and responsive to the needs of students, service providers, supervisors, rural health consumers, communities, and funders [12]. These intersecting expectations highlight how UDRHs can create value through place‐based training by aligning educational design with the practical realities of rural health systems and workforce contexts. Today's students expect flexible, digitally enhanced learning, while also seeking authentic, immersive experiences that prepare them for the complexities of rural practice. Health and community services require academic and practical strategies that minimise the burden on time‐limited supervisors and already stretched staff. Rural communities expect trust, continuity, and genuine partnerships with the expectation that training delivers benefits through local projects, service delivery and healthcare improvements, while funders expect high‐quality programs that translate into rural workforce intent and practice.

Health professional education must meet all these expectations while also accommodating multiple university and accrediting body objectives, such as integrating generalist and interprofessional practice skills, underpinned by cultural safety and authentic community engagement. Rural health services need practice‐ready graduates who can adapt to complex, resource‐limited environments and work effectively with diverse local populations [13, 14]. T raining should therefore reflect diverse service delivery models and prepare students for roles beyond the typical hospital and acute care environments and into aged care, mental health, disability and primary health care, while supporting the development of clinically relevant skills and practice readiness for complex rural contexts.

The quality of health professions training experiences is important [15]. A scoping review of rural placements identified four domains that underpin effective rural health professional education: learning and teaching in a rural context; placement characteristics such as length, continuity, and relevance; strong relationships between students, supervisors, and communities; and infrastructure such as housing and transport [16]. Professional development opportunities are important contributions as well. Continued investment in supervisor training and innovative models such as remote supervision remain essential [16]. Ongoing investment in local capacity and capability for high‐quality placements supports sustainable training models. Health professions training should also anticipate the realities and evolving conditions in rural areas, such as natural disasters and climate change, that are reshaping population health needs. Models of care such as Indigenous‐led services and the increasing role of digital health technologies as platforms for distributed models of care need to be integrated into teaching and research. The UDRHs have a history of leading teaching and placement innovation and driving change in the rural sector through locally tailored, place‐based initiatives [17, 18].

## 3 Place‐Based Research as Situated Knowledge Generation

Place‐based research contributes distinct value through the contextualisation and legitimisation of local knowledge and practice within rural health systems, including the exploration and articulation of the realities, priorities, and concerns of local people and communities. The creation and application of rural research validate effective practice and inform continuous improvement across health education, workforce planning, and service design. This research is important for effective education, workforce planning and service design. Likewise, it identifies factors that influence rural recruitment and retention, evaluates training outcomes, and guides investment in innovative and sustainable models of practice suited to rural contexts [19, 20]. Place‐based research also captures the complexities of practice in small and dispersed communities, contributing to ensuring that national health policy is informed by evidence drawn from rural experience [21]. Finally, approaches to, and insights gleaned from rural health research offer unique insights that have utility within metropolitan health settings.

The sustained presence of RHMT‐funded academic centres such as UDRHs has supported local partnerships, postgraduate supervision, and staff development, fostering a skilled rural research workforce. It has also facilitated the consistent use of robust research practices and methodologies that are contextualised to rural realities, informing both rigour and relevance. As a result, research has become part of the everyday culture of many rural health services, reinforcing quality improvement, workforce retention, and community engagement [22]. The growth in research capability contributes to tangible outcomes that guide health policy, inform best practice, and contribute to improved health outcomes for rural Australians [23].

Collaboration across the UDRH network continues to strengthen rural health research through nationally coordinated and regionally grounded initiatives [24]. Multi‐site studies provide the scale needed for generalisable findings for informing policy and national workforce strategies [25]. Locally led research allows for responsiveness to community priorities and the identification of practical solutions suited to local contexts [26]. Together, these complementary approaches create a research system that produces evidence capable of shaping national direction while remaining firmly anchored in local experience [27].

A distinctive strength of place‐based research is its capacity for genuine co‐production of knowledge with communities, health services, and First Nations partners, enabled by the embedded and sustained presence of UDRHs within rural communities, which supports locally informed priority‐setting and shared decision‐making in the research process. Indigenous‐led and community‐driven research undertaken through and alongside UDRHs, in partnership with local organisations and communities, demonstrates how culturally secure methodologies can deepen understanding of local health priorities while strengthening trust, participation, and self‐determination, thereby redistributing influence over research agendas towards community and First Nations partners. These approaches exemplify a unified but not uniform research system, one that values diversity of perspective while maintaining shared standards of quality, ethics, and impact. Recent analyses of UDRH activity show that these programs are bridging the urban–rural knowledge divide by producing research that is both rigorous and directly relevant to rural health priorities [22]. Sustaining a strong foundation of place‐based research allows the RHMT Program to continue advancing rural health education and to generate the evidence, innovation, and policy insight needed for ongoing improvement in workforce and service outcomes. In this way, the research arm of the program can strengthen not only local practice but also the academic integrity and national credibility of rural health as a field of study.

## 4 The Value of University Departments of Rural Health

Measuring the success of UDRHs by conventional, activity‐based outputs undervalues the mechanisms through which place‐based education and research contribute to distinct outcomes and system‐level value. Beyond their teaching and research functions, UDRHs influence community capital in their regions, influencing its forms of human, social, cultural, political, financial, and built capital [24, 28]. They offer a distinct contextualised approach that is grounded in a commitment to working *in, with and for* the rural communities they serve. Viewed collectively, UDRH place‐based education and research are associated with enhanced community resilience and economic participation, reinforcing Australia's capacity to deliver equitable healthcare across geography and culture [24]. UDRHs are linked to regional community development, reaffirming their strategic role within the RHMT Program [24]. Integrated, place‐based infrastructure and staffing provide the platform from which future rural health training, research, and workforce initiatives can continue to develop. Extending the role of the UDRHs to support early‐career rural graduates (e.g., through structured work transition support, access to professional development and mentorship) as an enhancement within the existing RHMT framework rather than as a separate program model, could enhance the program's impact by targeting strategies known to influence retention, including building connection with community through ongoing professional development [6].

## 5 Conclusion

The RHMT Program is a cornerstone of Australia's rural health policy, facilitating sustained benefits across education, workforce, research, and community development. Through continued collaboration across their networks and communities, the UDRHs are well positioned to play a central role in supporting the ongoing development of place‐based training and research that contributes to strengthening the rural health workforce.

## Author Contributions

**Leanne Brown:** conceptualization, writing – original draft, writing – review and editing, resources. **Joanne Hutchinson:** conceptualization, writing – original draft, writing – review and editing, resources. **James Debenham:** conceptualization, writing – original draft, writing – review and editing, resources. **Pam Harvey:** conceptualization, writing – original draft, writing – review and editing, resources.

## Funding

The authors would like to acknowledge Australian Government’s Rural Health Multidisciplinary Training (RHMT) funding in enabling this statement.

## Ethics Statement

The authors have nothing to report.

## Conflicts of Interest

The authors declare no conflicts of interest.

## Data Availability

The authors have nothing to report.
